# Citrate regional anticoagulation of 500 ml/min of extracorporeal blood flow: an experimental study

**DOI:** 10.1186/s40635-025-00771-7

**Published:** 2025-06-24

**Authors:** Sebastiano Maria Colombo, Luigi Vivona, Michele Battistin, Vittorio Scaravilli, Alessandro Galli, Chiara Anzanello, Elisa Cipriani, Francesca Gori, Serena Todaro, Carlo Valsecchi, Daniele Dondossola, Anna Paola Marcello, Andrea Carlin, Antonio Pesenti, Giacomo Grasselli, Alberto Zanella

**Affiliations:** 1 Department of Anesthesia, Critical Care and Emergency, Fondazione IRCCS Ca’ Granda Ospedale Maggiore Policlinico, Via Della Commenda 16, 20122 Milan, Italy; 2 Department of Anaesthesia and Intensive Care, IRCCS MultiMedica San Giuseppe Hospital, Milan, Italy; 3 Center for Preclinical Research, Fondazione IRCCS Ca’ Granda Ospedale Maggiore Policlinico, Milan, Italy; 4 Department of Biomedical, Surgical and Dental Sciences, University of Milan, Milan, Italy; 5 Dipartimento Emergenza, Urgenza e Area Critica, ASST Papa Giovanni XXIII, Bergamo, Italy; 6 Department of Pathophysiology and Transplantation, University of Milan, Milan, Italy; 7 Intensive Care Unit and Regional ECMO Referral Center, Careggi University Hospital, Florence, Italy; 8 Liver Transplant and General Surgery Unit, Fondazione IRCCS Ca’ Granda Ospedale Maggiore Policlinico, Milan, Italy; 9 Department of Medicine, Hematology-Fisiopatologia Delle Anemia, Fondazione IRCCS Ca’ Granda Ospedale Maggiore Policlinico, Milan, Italy

**Keywords:** Citrate, Regional anticoagulation, Ion-exchange resins, 500 mL/min

## Abstract

**Background:**

Regional citrate anticoagulation (RCA) is the most widespread technique which allows to perform extracorporeal treatments, avoiding the complications of systemic anticoagulation. Due to limited citrate clearance, RCA may be applied only to low extracorporeal blood flows (i.e., BF < 200 ml/min). In this proof of concept study, we developed an innovative RCA technique based on Ion Exchange Resin (i-ER) technology capable of regionally anticoagulating BF up to 500 mL/min.

**Methods:**

Six healthy swine (41.0 ± 3.1 kg) were sedated, mechanically ventilated, and connected to a prototype extracorporeal circuit for continuous renal replacement therapy featuring a citrate-removal stage based on absorbent materials and replacement fluids. Blood flow was 500 ml/min. Sodium citrate was continuously infused at the circuit inlet (5 mmol/L). Heparin was continuously infused. Citrate concentration and Kaolin Heparinase thromboelastography (KH-TEG) were measured on arterial blood, extracorporeal blood downstream the citrate infusion port, and downstream the citrate-removal stage. Samples were collected at baseline, 2, 8, 15, 30, 45, 60, 90, and 120 min for citrate and at baseline, 2, 30, 60, and 120 min for KH–TEG. Calcium chloride was infused to maintain systemic ionized calcium within the physiological range. The experiment lasted 2 h.

**Results:**

During the whole experiment, KH–TEG in the artery showed normal coagulation: reaction time (R) was 8.30[6.80–10.10] min, with Maximum Amplitude (MA) of 71.70[67.90–77.00] mm, while in the extracorporeal circuit, KH–TEG showed no sign of clot formation R > 60 min, MA = 0 mm. Citrate concentrations in blood samples were stable within 30 min, then slowly increased. The efficacy of the citrate-removal dropped from 93.8 ± 3.4% to 48.3 ± 1.5% at the beginning (2 min) and at the end (2 h), respectively (*p* < 0.001), due to loss of efficiency of the iERs.

**Conclusions:**

This study demonstrates that iER-based RCA is a feasible and effective technique for regional anticoagulation of extracorporeal blood flow up to 500 mL/min for 60 min without significant complications.

**Supplementary Information:**

The online version contains supplementary material available at 10.1186/s40635-025-00771-7.

## Introduction

Blood anticoagulation is a mainstay of any extracorporeal blood treatment [1]. With the current artificial surface technology, extracorporeal blood treatment without anticoagulation inevitably leads to both circuit and systemic thromboembolic complications [2, 3]. Systemic heparin infusion is the most widespread method to achieve anticoagulation [4, 5]. Unfortunately, this approach is fraught with possible severe and even lethal hemorrhage [6, 7]. To reduce these complications, several regional coagulation techniques have been developed [8, 9], regional citrate anticoagulation (RCA) being the most employed [10]. Citrate infused in the extracorporeal circuit chelates calcium, thus inhibiting the clotting cascade and platelet activation. Then, once in the systemic circulation, citrate is cleared by liver and kidney metabolism. [11, 12] Despite being safe and effective in several settings [13], due to the limited citrate systemic clearance [14, 15], RCA can be employed only for extracorporeal blood treatment with low blood flow (BF), i.e., lower than 200 mL/min if continuous renal replacement therapy is applied. Whether employed with higher BF, RCA may lead to citrate net overload or accumulation, causing acid–base and calcium derangements [16–23].

We developed an innovative RCA technique based on Ion Exchange Resin (i-ER) [24] technology capable of regionally anticoagulating BF up to 500 mL/min to overcome such issues. I-ERs are insoluble polymers with acidic or basic functional groups capable of exchanging cations or anions within the surrounding aqueous solution [25, 26]. Integrating an anionic i-ER in the extracorporeal circuit theoretically allows for citrate clearance and thus limits citrate systemic accumulation.

The primary aim of this proof of concept study is to evaluate in a large-animal experiment the feasibility, efficacy, and short-term safety of this innovative i-ER-based RCA in anticoagulating high extracorporeal blood flow (i.e., 500 mL/min).

## Materials and methods

### Animal care and ethical approval

This study was approved and conducted according to the Institutional Guidelines for the Care and Use of Laboratory Animals. The Italian Ministry of Health approved the study (1178/2020-PR), and animals were treated according to international recommendations [27].

### Animal preparation and management

Complete descriptions of animal care, animal preparation and instrumentation, animal monitoring, and management are reported in the online supplement and our previous experiments [26, 28, 29].

Following a parenteral injection of 150 IU/kg of unfractionated heparin (UFH) (Epsoclar, Pfizer Srl, Latina, Italy), the right iliac vein and the right external jugular vein were cannulated (Single stage venous cannula 18 Fr, 25 cm, Medtronic, Minneapolis, MN) and connected to a custom-made extracorporeal circuit optimized for i-ER-based RCA. A continuous infusion of UFH was provided, targeting an activated clotting time > 300 s (GEM PCL Plus, Instrumentation Laboratory SpA Werfen, Milano, Italy).

Given that this was an exploratory proof-of-concept study using large animals, we administered systemic anticoagulation by heparin infusion throughout the 2-h experiment (maintaining an ACT > 300 s, according to our group previous experiments [26, 28–30]) to prevent clot formations during other phases other than the iER-based RCA evaluation. Consequently, thromboelastographic analyses were conducted using kaolin–heparinase cuvettes, which ensured that the heparin infusion did not influence the interpretation of our results.

Throughout the experiment, arterial ionized calcium (iCa) was kept within 1.2 and 1.3 mmol/L while potassium (K^+^) within 3.5 and 4.5 mmol/L by a parenteral infusion of replacement solutions (calcium chloride 0.68 mmol/mL, potassium chloride 2 mmol/mL). At the end of the experiment, animals were euthanized while still under deep sedation, with an injection of 40 mEq of potassium chloride.

### Extracorporeal circuit

**Fig. 1:**
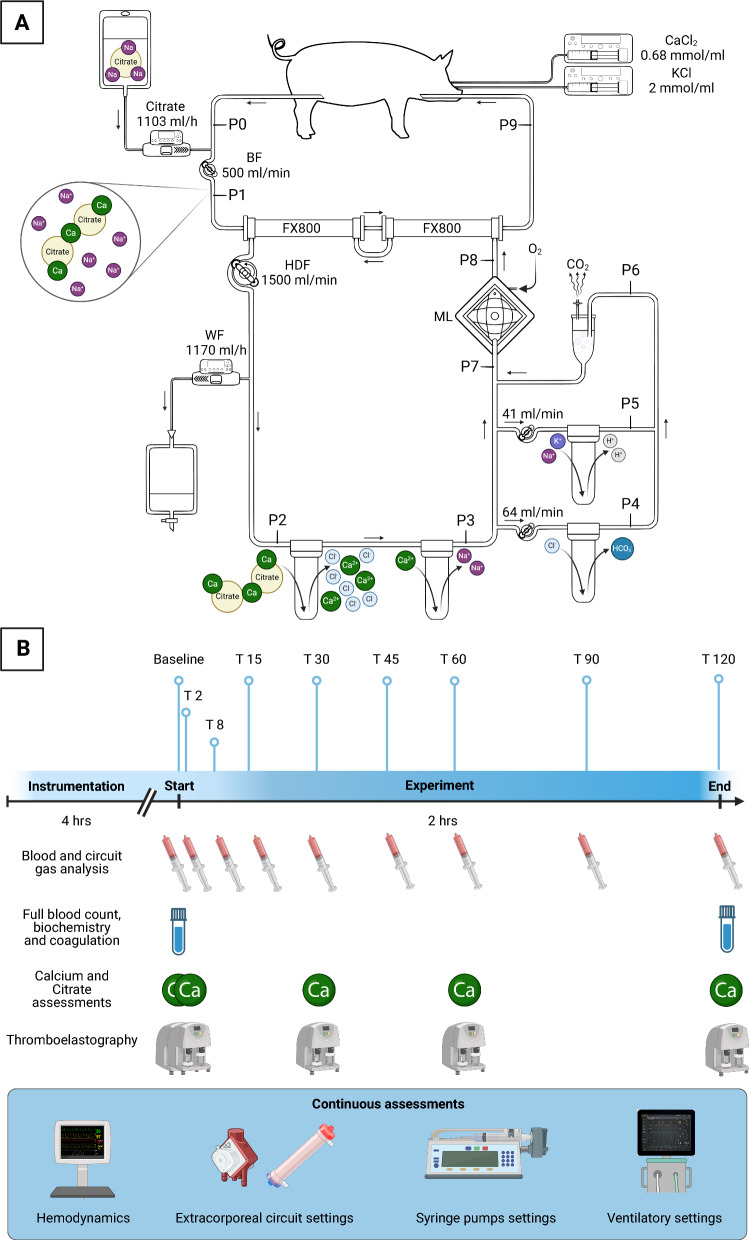
Extracorporeal circuit (**A**) and experiment timeline (**B**). Details in the text. Created in BioRender. Colombo, S. (2025) https://BioRender.com/v91y000

A schematic representation of the custom-made extracorporeal circuit is shown in Fig. 1A. It was composed of a blood circuit and a hemodiafiltrate circuit. First, blood was drained from the right iliac vein with a peristaltic pump (Multiflow Roller Pump Module H10 series, Stöckert Shiley, Munich, Germany) called *blood pump*, then flowed through two hemodiafilters (FX800, Fresenius Medical Care, Bad Homburg, Germany) and was reinfused into the right external jugular vein. Two hemodiafilters in series were used to provide adequate dialysis. Blood flow (BF) was kept constant at 500 mL/min.

After the sampling point of the drainage blood (Blood inlet, P0 in the circuit), a Sodium Citrate infusion (Sodium Citrate 4%, Fresenius Kabi AG, Bad Homburg, Germany at 1103 mL/h by Volumat MC Agilia, Fresenius Kabi, Le Grand Chemin, France) was administered to achieve citrate concentration of 5 mmol/L before the hemodiafilters (pre-filter blood, P1 in the circuit).

After the extracorporeal treatment, the blood returned to the pig (Blood outlet, P9 in the circuit).

In the hemodiafiltration circuit, the outlet and the inlet ports of the hemodiafilters were connected to form a closed-loop circuit. The hemodiafiltrate was then directed, countercurrent to the blood, through a series of cartridges (FCR 35, Nobel, Segrate, Italy), containing 600 ml of ion exchange resins each, using a peristaltic pump (Multiflow Roller Pump Module H10 series), referred to as the *hemodiafiltrate pump*, at a flow rate of 1500 mL/min (Hemodiafiltrate flow, HDF):

- In the first portion of the hemodiafiltrate circuit, three ion exchange resins were connected in series. The first two were anionic i-ERs, while the third was a cationic i-ER (high capacity, mixed matrix PMB 101–3 polystyrene and divinylbenzene, Pure Resin, HSEDA Shangyu, Zhejiang, China). Two withdrawal ports were positioned before and after these i-ERs (P2 and P3). This first portion of i-ER exchanges the citrate anion with the chloride ion, consequently releasing the ionized calcium chelated by the citrate. Calcium is then exchanged with sodium to maintain effective anticoagulation.
- In the second portion of the hemodiafiltrate circuit, two couples of ion exchange resins were connected using a specific one-to-one parallel circuit to provide ions equilibrium. Specifically, a portion of the hemodiafiltrate was diverted across anionic resins at a flow rate of 64 mL/min, while another 41 mL/min of hemodiafiltrate was diverted into cationic resins. Three withdrawal ports were positioned after the anionic resin (P4), after the cationic resin (P5), and after the rejoining of these (P6). This second portion of resin circuit, placed in parallel along the primary hemodiafiltration circuit, rebalances the chloride and sodium ions (previously exchanged in the first portion of the hemodiafiltrate circuit) with bicarbonate and hydrogen ions, respectively. These ions form carbonic acid, which breaks down into water and carbon dioxide, subsequently eliminated as a volatile gas. The reactions across the second section of the hemodiafiltration circuit are exothermic and produce a significant amount of carbon dioxide. For this reason, a gas trap was placed immediately after rejoining the two paths to limit gas entry into the primary hemodiafiltrate circuit.

At the end of the hemodiafiltrate circuit, a membrane lung (ML–Quadrox–iD Adult, Maquet Cardiopulmonary GmbH, Rastatt, Germany) was inserted. The ML removes the CO_2_ excess generated by the reaction between the H^+^ and HCO₃^⁻^ ions released by the exchange resins before the ML, acting also as a bubble trap. This ML was ventilated with a constant flow of pure oxygen (10 L/min), and the temperature was kept at 38 ºC throughout the experiment to avoid coagulation disorders. In addition, two withdrawal ports were placed before and after the ML (P7 and P8 in the circuit).

Along the hemodiafiltrate circuit, 1170 mL/h of the hemodiafiltrate (Waste Flow, WF) was diverted before the first ion exchange resin and then discarded by a peristaltic pump (Volumat MC Agilia), referred to as *waste pump*. This flow ensured the pig remained euvolemic.

### Ion exchange preparation

The ion exchange resin cartridges were washed and rinsed with specific solutions before use. See the Online Supplement for further details. The entire circuit was finally primed with Bicarbonate Buffered Haemofiltration Solution 4 mmol/L potassium (MultiBic, Fresenius Medical Care, Bad Homburg, Germany) both in the blood circuit (priming volume ≈ 1 L) and in the hemodiafiltrate circuit (priming volume ≈ 1.5 L).

### Experimental design and measurements

Upon completion of the instrumentation, the feasibility and safety of the i-ER-based RCA technique were evaluated in a 2-h proof-of-concept experiment.

Nine different steps were defined according to specific timepoints: baseline (only blood flow, without i-ER-based RCA), T2 (2 min), T8 (8 min), T15 (15 min), T30 (30 min), T45 (45 min), T60 (60 min), T90 (90 min), T120 (120 min).

Hemodynamics (i.e., heart rate, arterial pressures, core temperature, and oxygen peripheral saturation), ventilatory parameters (i.e., minute ventilation, respiratory rate, tidal volume, peak pressure, plateau pressure, and mean airway pressure), and circuit settings (i.e., flows and pressures) were recorded. Refer to Fig. 1B for a schematic timeline of the sample collection.

At each step, arterial and circuit port samples were withdrawn for gas analyses and ion concentrations (Ca^++^, Na^+^, and Cl^−^) (ABL 800 gas analyzer; Radiometer, Copenhagen, Denmark). In addition, laboratory tests were conducted at specific timepoints to assess magnesium and total calcium concentrations (ADVIA Chemistry, Siemens Healthcare Diagnostics Inc, Tarrytown, NY), fibrinogen level according to Clauss method (ACL TOP 700 CTS, Instrumentation Laboratory, Bedford, MA), and complete blood count (XT-2000i, Sysmex, Kobe, Japan). Furthermore, total proteins, hemolysis indicators like lactate dehydrogenase (ADVIA Chemistry, Siemens Healthcare Diagnostics Inc), and plasma free hemoglobin concentration were also measured after instrumentation as well as at the end of the study. Further details can be found in the Online Supplement.

To evaluate the anticoagulation efficacy, blood was sampled from the arterial line (ART), from blood-inlet (P1), and the blood outlet (P9) and subsequently evaluated by viscoelastic coagulation tests (TEG® 5000, Haemonetics Corporation, Braintree, MA, US). To evaluate coagulation independently from the level of systemic heparinization and the potential bias due to heparin’s continuous intravenous infusion, the Kaolin–Heparinase test technique (KH–TEG) was used. The following KH–TEG parameters were studied: reaction time (R), kinetics time (K), $\alpha$ angle ($\alpha$**)**, maximum amplitude (MA), and clot lysis 30 min after MA (Ly30). Samples with R > 60 min were deemed unclotted; thus, the KH–TEG tests were interrupted. Consequently, values of 60 min for R and K and 0 for $\alpha$ angle, MA, and Ly30 were assigned arbitrarily.

Plasmatic citrate concentration was evaluated throughout the experiment (Citrate evaluation kit, Sigma Aldrich, St. Louis, MO, USA). The efficiency of citrate removal by iERs was calculated using the ratio between the difference in pre-filter blood citrate concentration (P1) and Blood outlet citrate concentration (P9) with the pre-filter blood citrate concentration (P1), ([CitrateP1]–[CitrateP9]/[CitrateP1]).

### Statistical analysis

The sample size was based on our experience from previous experiments.

When appropriate, data are presented as mean ± standard deviation (SD) or median and interquartile range [IQR]. A two-way analysis of variance for repeated measures (two-way RM–ANOVA) was performed using a residual maximum likelihood method to fit a general linear model. Circuit withdrawal port (i.e., nine levels) and time (Baseline to T120) were considered fixed factors, while animals were considered random effects. Post-hoc Student *t* test with Tukey adjustment was used for multiple comparisons. Two-tailed values of P below 0.05 were considered statistically significant. Statistical analyses were performed using the JMP Pro 16 statistical software (SAS, Cary, NC, US), GraphPad Prism 10 for Mac (GraphPad Software, San Diego, CA, USA).

## Results

All the 6 animals (41.00 ± 3.14 kg) completed the study without significant complications.

### Regional anticoagulation

The systemic and extracorporeal KH–TEG parameters are reported in Table 1. The i-ER-based RCA technique provided efficient blood anticoagulation throughout the entire extracorporeal circuit, while systemic coagulation remained stable and unaffected.

**Table 1:** Thromboelastographic parameters

		Baseline	T2	T30	T60	T120	*p* value
R (min)	ART	9.10 [6.35–10.13]	11.95 [7.80–16.10]	8.90 [6.90–10.35]	8.20 [6.30–10.45]	8.10 [6.25–10.80]	0.5581
	P1	–	60.00 [60.00–60.00]	60.00 [60.00–60.00]	60.00 [60.00–60.00]	60.00 [60.00–60.00]	1.0000
	P9	–	60.00 [60.00–60.00]	60.00 [60.00–60.00]	60.00 [60.00–60.00]	60.00 [60.00–60.00]	1.0000
K (min)	ART	2.30 [1.55–3.25]	4.20 [1.80–6.60]	2.30 [1.95–3.30]	2.10 [1.70–3.70]	2.30 [1.55–3.40]	0.4602
	P1	–	0.00 [0.00–0.00]	0.00 [0.00–0.00]	0.00 [0.00–0.00]	0.00 [0.00–0.00]	1.0000
	P9	–	0.00 [0.00–0.00]	0.00 [0.00–0.00]	0.00 [0.00–0.00]	0.00 [0.00–0.00]	1.0000
$\alpha$(°)	ART	64.80 [52.25–73.80]	50.65 [32.00–69.30]	61.40 [55.20–66.60]	65.80 [49.65–69.00]	62.40 [41.10–65.20]	0.4313
	P1	–	0.00 [0.00–0.00]	0.00 [0.00–0.00]	0.00 [0.00–0.00]	0.00 [0.00–0.00]	1.0000
	P9	–	0.00 [0.00–0.00]	0.00 [0.00–0.00]	0.00 [0.00–0.00]	0.00 [0.00–0.00]	1.0000
MA (mm)	ART	73.40 [68.33–80.13]	66.25 [56.50–76.00]	71.70 [62.85–75.50]	69.00 [63.40–76.15]	72.10 [70.75–77.30]	0.3907
	P1	–	0.00 [0.00–0.00]	0.00 [0.00–0.00]	0.00 [0.00–0.00]	0.00 [0.00–0.00]	1.0000
	P9	–	0.00 [0.00–0.00]	0.00 [0.00–0.00]	0.00 [0.00–0.00]	0.00 [0.00–0.00]	1.0000
Ly30 (%)	ART	1.5 [0.5–26.9]	–	0.4 [0.1–0.7]	0.2 [0.1–3.2]	1.7 [0.8–3.2]	0.7109
	P1	–	0.0 [0.0–0.0]	0.0 [0.0–0.0]	0.0 [0.0–0.0]	0.0 [0.0–0.0]	1.0000
	P9	–	0.0 [0.0–0.0]	0.0 [0.0–0.0]	0.0 [0.0–0.0]	0.0 [0.0–0.0]	1.0000

Data are reported as median [IQR] or mean ± SD

Samples obtained from the Arterial line (ART), after sodium–citrate infusion in the circuit (P1) and blood outlet (P9). Only heparinase results are reported

Statistical analysis: No statistically differences between timepoints were observed

R, reaction time; K, K-time; $\alpha$, angle; MA, maximum amplitude; Ly30, % of lysis 30 min after MA

Specifically, R-time never showed any sign of clot formation in the pre-filter blood (P1) or in the blood outlet (P9), while normal values were observed in the arterial blood (overall median R-time: 60.00[60.00–60.00] vs. 8.30[6.80–10.10] min; *p* < 0.001).

Furthermore, MA-amplitude was stable at 73.40[68.33–80.13] and 72.10[70.75–77.30] at baseline and end-experiment, respectively (*p* = 0.4812); same trends have been observed in K (2.30[1.55–3.25] vs. 2.30[1.55–3.40], *p* = 0.4602), α (64.80[52.25–73.80] vs. 62.40[41.10–65.20], *p* = 0.4313) and Ly30 (1.5[0.5–26.9] vs. 1.7[0.8–3.2], *p* = 0.7109).

No significant alterations in platelets and fibrinogen systemic concentrations were observed (Table 2).

**Table 2:** Laboratory results

	Baseline	T120	*p* value
Alb (g/dL)	2.35 [2.22–2.57]	2.35 [2.10–2.62]	0.8220
AST (IU/L)	25 [17–26]	30 [24–34]^&^	0.0121
ALT (IU/L)	44 [40–61]	48 [40–59]	0.6756
BilT (mg/dL)	0.09 [0.08–0.11]	0.13 [0.07–0.28]	0.1271
CPK (IU/L)	592 [454–975]	526 [392–839]	0.2285
Crea (mg/dL)	0.73 [0.67–0.79]	0.69 [0.58–0.75]	0.0895
D-dim (mcg/L)	355 [136–565]	288 [212–443]	0.3460
Plt (10^3^/$\mu$ L)	316.0 ± 137.4	348.2 ± 141.2	0.7030
Fib (mg/dL)	111.0 [92.5–135.5]	114.0 [107.5–149.0]	0.1899
Hb-free (mg/dL)	4.40 [3.30–8.12]	6.90 [4.75–8.25]	0.4034
LDH (IU/L)	395.5 [376.7–424.5]	380.0 [345.0–497.5]	0.8126
Tot Prot (g/dL)	4.95 [4.30–5.50]	4.50 [4.45–5.30]	0.5467
Urea (mg/dL)	8.00 [5.75–9.75]	9.00 [7.50–11.50]	0.0905

Data are reported as median [IQR] or mean ± SD

Statistical analysis: ^&^*p* < 0.05 vs. Baseline

Alb, albumin; AST, aspartate transaminase; ALT, alanine transaminase; BilT, total bilirubine; CPK, creatine phosphokinase; Crea, creatinine; D-dim, D-dimers; Plt, Platelets; Fib, Fibrinogen; Hb-free, plasma-free hemoglobin; LDH, lactate dehydrogenase; Tot Prot, total proteins

The i-ER-based RCA technique significantly reduced ionized calcium concentrations (iCa) in the pre-filter blood (P1) and blood outlet (P9) compared to the arterial blood.

**Fig. 2:**
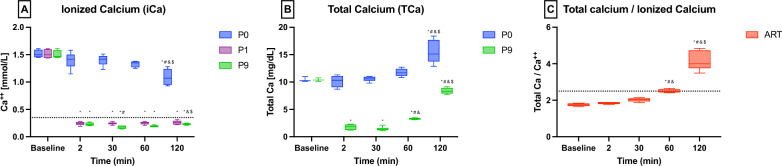
Total and ionized calcium concentrations. Data are reported as median [IQR]. Samples obtained from the Arterial line (ART), blood inlet (P0), after sodium–citrate infusion in the circuit (P1) and blood outlet (P9). **A** Ionized Calcium concentration (iCa) along the extracorporeal circuit. **B** Total Calcium concentration (TCa) along the extracorporeal circuit. **C** TCa:iCa ratio in the arterial blood. Statistical analysis: *p < 0.05 vs. Baseline, ^#^*p* < 0.05 vs. T2, and *p* < 0.05 vs. T30, ^$^*p* < 0.05 vs. T60. iCa. Ionized calcium; TCa. Total calcium

iCa dropped from 1.38 ± 0.10 mmol/L in the arterial blood to 0.24 ± 0.02 mmol/L in the pre-filter blood (P1) and 0.19 ± 0.02 mmol/L in the blood outlet (P9) at the beginning of the i-ER-based RCA treatment (iCa at 15 min: *p* < 0.001). The concentrations of iCa in the blood inlet—before the extracorporeal treatment with RCA (P0)—were comparable to the systemic concentrations (iCa at 15 min: ART 1.38 ± 0.10 mmol/L vs. P0 1.41 ± 0.10 mmol/L, *p* = 0.5969). The effects of the passage of blood through the extracorporeal circuit on iCa are shown in Fig. 2A.

The effects of the i-ER-based RCA technique on total calcium concentrations (TCa) are presented in Fig. 2B. TCa was stable up to 60 min of treatment and subsequently progressively increased both in systemic and extracorporeal samples. Systemic TCa increased from 10.3 [10.1–10.8] mg/dL at the beginning (15 min) to 15.5[15.1–18.2] mg/dL at the end of treatment (*p* < 0.0001). As for iCa, TCa concentrations at the blood inlet (P0) were comparable to arterial concentrations during the entire experiment. (TCa at 15 min: ART 10.1[9.9–10.8] mg/dL vs. P0 10.3[10.0–10.7] mg/dL; *p* = 0.7752).

During the second hour of the experiment, a dramatic increase in the TCa:iCa ratio was observed. The ratio was below 2.5 until 60 min of treatment, then almost doubled at the end of the experiment (T60 2.48[2.44–2.59] vs. T120 4.00[3.77–4.73]; *p* < 0.001) (Fig. 2C).

**Fig. 3:**
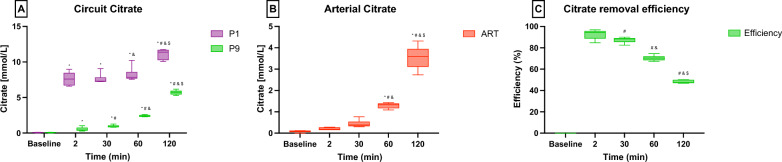
Citrate concentrations and removal efficiency. Data are reported as median [IQR]. Samples obtained from the Arterial line (ART), after sodium–citrate infusion in the circuit (P1) and blood outlet (P9). **A** Citrate concentration along the extracorporeal circuit. **B** Citrate concentration in the arterial blood. **C** Citrate removal efficiency. Statistical analysis: *p < 0.05 vs. Baseline, ^#^*p* < 0.05 vs. T2, and *p* < 0.05 vs. T30, ^$^*p* < 0.05 vs. T60

Citrate concentrations significantly changed along the extracorporeal circuit. Looking at the first 60 min of treatment, citrate dropped from a median value of 7.85[7.69–8.58] mmol/L in pre-filter blood (P1) to 2.38[2.32–2.50] mmol/L in blood outlet (P9) due to the i-ER-based RCA treatment (*p* < 0.001) (Fig. 3A). In addition, direct systemic accumulation of citrate was observed since the first hour of the experiment, with arterial citrate increasing from 0.20[0.17–0.32] mmol/L at 15 min to 3.60[3.11–3.94] mmol/L at 2 h (*p* < 0.001) (Fig. 3B).

The efficiency reduction in citrate removal by i-ER-based RCA circuit can be observed in Fig. 3C. Citrate removal dropped from 93.8 ± 3.4% to 48.3 ± 1.5% at the beginning (15 min) and at the end (2 h), respectively (*p* < 0.001).

For further details on regional anticoagulation, iCa and TCa concentrations, and citrate concentration, see Table S1 in the Online Supplement.

### Electrolytes and acid–base balance

Sodium remained almost constant during the entire first hour of the experiment, while a significant decrease was observed in the last hour. As reported in Table 3, systemic Na^+^ decreased from 138.2 ± 2.6 mmol/L to 132.7 ± 4.1 mmol/L at baseline and T120, respectively (*p* = 0.0133).

**Table 3:** Arterial blood gas results

	Baseline	T15	T30	T60		T120		*p* value
pH	7.46 ± 0.03	7.47 ± 0.07^&^	7.52 ± 0.04^&^	7.54 ± 0.03^&$^		7.55 ± 0.04^&$^		< 0.0001
pCO_2_ (mmHg)	41.0 ± 2.9	40.6 ± 6.5	37.9 ± 2.0	37.2 ± 2.0		35.7 ± 3.1^&$^		0.0152
pO_2_ (mmHg)	182.0 ± 27.0	174.5.0 ± 23.8	175.8 ± 16.8	175.2 ± 19.3		169.2 ± 19.5		0.3966
Hb (g/dL)	8.0 ± 1.0	8.1 ± 1.0	8.3 ± 0.9	7.9 ± 0.9		7.8 ± 1.9		0.6513
O_2_Hb (%)	97.7 ± 0.4	97.7 ± 0.5	97.6 ± 0.3	97.6 ± 0.3		97.8 ± 0.7		0.3659
Hct (%)	25.0 ± 2.9	25.2 ± 3.0	25.8 ± 2.9	24.5 ± 2.9		24.2 ± 5.8		0.6478
K^+^ (mmol/L)	3.9 ± 0.3	4.2 ± 0.3	4.0 ± 0.3	4.0 ± 0.2		3.9 ± 0.2		0.0541
Na^+^ (mmol/L)	138.2 ± 2.6	137.7 ± 2.3	136.5 ± 2.1	135.3 ± 1.9		132.7 ± 4.1^&$^		0.0041
Cl^−^ (mmol/L)	100.8 ± 4.8	104.3 ± 3.4	102.8 ± 2.8	98.7 ± 2.3		94.2 ± 8.6^$#^		0.0029
HCO3^−^ (mmol/L)	28.7 ± 1.8	29.2 ± 1.1	31.0 ± 1.6	31.6 ± 1.3^&^		31.3 ± 4.3		0.0113
Lac (mmol/L)	2.1 ± 0.7	2.2 ± 3.4	2.0 ± 0.4	2.2 ± 0.4		2.5 ± 0.6		0.0812
Glu (mg/dL)	128.0 ± 14.3	113.3 ± 32.8	123.7 ± 15.7	123.8 ± 14.4		117.7 ± 13.6		0.7491
BE (mmol/L)	4.9 ± 2.0	5.3 ± 2.0	7.2 ± 1.6	8.1 ± 1.5		7.8 ± 4.6		0.2224
P (mg/dl)	8.05 ± 0.66	5.52 ± 0.57^&^	5.70 ± 0.57^&^	5.97 ± 0.46^&^		6.47 ± 0.47^&$#^		< 0.0001
Mg (mg/dl)	1.67 [1.58–1.74]	1.07 [1.04–1.16]^&^	1.04 [1.01–1.11]^&^	1.11 [1.09–1.19]^&^		1.28 [1.19–1.36]^&$#@^		< 0.0001
iCa (mmol/L)	1.48 ± 0.04	1.38 ± 0.10	1.28 ± 0.09^&^	1.16 ± 0.07^&$^		1.00 ± 0.17^&$#^		< 0.0001
TCa (mg/dL)	10.3 [10.1–10.8]	10.1 [9.9–10.8]	10.4 [10.1–10.6]	11.5 [11.0–12.5]		15.5 [15.1–18.2]^&$#@^		< 0.0001

Data are reported as median [IQR] or mean ± SD

Samples obtained from the Arterial line

Statistical analysis: ^&^*p* < 0.05 vs. Baseline, ^$^*p* < 0.05 vs. T15, ^#^*p* < 0.05 vs. T30, ^@^*p* < 0.05 vs. T60

pCO_2_, arterial carbon dioxide pressure; pO_2_, arterial oxygen pressure; Hb, hemoglobin; O_2_Hb, hemoglobin oxygen saturation; Hct, hematocrit; K^+^, potassium; Na^+^, sodium; Cl^−^, chloride; HCO3^−^, bicarbonates; Lac, lactate; Glu, glucose; BE, base excess; P, phosphorus; Mg, magnesium; iCa, ionized calcium (Ca^++^); TCa, total calcium

Systemic concentrations of potassium remained stable during the experiment (3.9 ± 0.3 mmol/L vs. 3.9 ± 0.2 mmol/L at baseline and T120, respectively; *p* = 0.3141, Table 3).

Arterial chloride dropped from 100.8 ± 4.8 mmol/L to 94.2 ± 8.6 mmol/L at baseline and T120, respectively (*p* = 0.0135) (Table 3).

Magnesium systemic concentration decreased from 1.67[1.58–1.74] mg/dL to 1.28[1.19–1.36] mg/dL at baseline and T120, respectively (*p* < 0.001) (Table 3).

Phosphorus concentrations decreased over the 2-h experiment, with arterial concentrations dropping from 8.05 ± 0.66 mg/dL to 6.47 ± 0.47 mg/dL at baseline and T120, respectively (*p* < 0.001) (Table 3).

Acid–base regulation was also affected due to electrolyte exchanges along the circuit during the i-ER-based RCA treatment. As a result, arterial pH increased from 7.46 ± 0.03 to 7.55 ± 0.04 at baseline and T120, respectively (*p* < 0.001) (Table 3).

A complete summary of all electrolyte concentrations and acid–base parameters in the extracorporeal blood along the circuit is presented in Tables S2–S10 in the Online Supplement.

### Systemic safety

The hemodynamics and ventilatory parameters were stable throughout the study, as shown in Table S5 in the Online Supplement. No significant adverse events were observed in the pigs during the study.

Blood gas and laboratory results obtained during the experiment are presented in Tables 2 and 3. No significant changes in values were observed, except for the electrolytes and pH as described earlier. Free hemoglobin levels showed a slight increase from 5.31 ± 2.42 mg/dL to 6.58 ± 2.25 mg/dL at baseline and T120, respectively (*p* = 0.4034).

## Discussion

We have evaluated an innovative RCA technique based on i-ERs technology for anticoagulating extracorporeal blood flow up to 500 mL/min. In a large animal model of healthy swine connected to a custom-made extracorporeal circuit, the i-ER-based RCA proved to be feasible and effective in reducing calcium concentrations and removing citrate, thus achieving regional anticoagulation, as confirmed by the KH–TEG analysis while avoiding systemic citrate accumulation.

Due to the activation of the coagulation cascade upon contact between blood and extracorporeal surfaces, blood anticoagulation is necessary during extracorporeal blood treatments [2, 3, 5, 7]. However, systemic anticoagulation can be associated with severe and even fatal complications, such as massive bleeding and intracranial hemorrhage, even at low doses of unfractionated heparin [6, 7]. Therefore, the i-ER-based RCA provides a promising alternative for anticoagulation during extracorporeal blood treatments, offering effective regional anticoagulation while avoiding systemic complications.

In recent decades, several techniques have been proposed to overcome the complications associated with systemic anticoagulation during extracorporeal treatments, including dilutional approaches, regional heparin–protamine infusions, prostaglandin infusion, and variations in materials and blood flows [8, 31–34]. However, citrate anticoagulation remains the most widely used regional blood anticoagulation worldwide [10]. It is the preferred anticoagulation method for continuous renal replacement therapy due to its superior performance in filter life span and total delivered therapy compared to systemic heparin infusion [13, 35]. Citrate is infused directly into the extracorporeal circuit at the blood inlet and, chelating calcium, reduces its concentration, thus providing immediate anticoagulation of blood [11, 36, 37]. Roughly 50% of the resulting citrate–calcium complexes are eliminated via continuous renal replacement therapy, while the remaining portion enters the systemic circulation, where it is metabolized by the liver [11, 38]. The calcium lost through this process is then replaced in the blood outlet, using automated algorithms to calculate the required amount [39, 40]. Unfortunately, citrate clearance is limited, so only extracorporeal BF up to 200 ml/min can be anticoagulated by citrate infusion. In addition, accurately estimating calcium losses can be challenging, even with the use of automated systems that still require operator-dependent interventions [41–43]. Consequently, several complications have been reported during RCA including citrate accumulation, acid–base imbalances, and electrolyte disorders [16–22]. Moreover, citrate infusion contributes to caloric load, which can be detrimental, especially for critically ill patients [23, 44].

We previously described an alternative technique for regional anticoagulation that addressed some of the limitations associated with citrate-based anticoagulation. Our technique used cation i-ERs to reduce the concentration of calcium and achieve anticoagulation without the need for citrate infusion. In our initial report, we demonstrated the feasibility of this approach, but a high recirculation of calcium-free ultrafiltrate was necessary, which limited its application to extracorporeal blood flows lower than 150 mL/min [26]. To overcome some of the limitations of our previous technique and of existing citrate-based anticoagulation technologies, we designed and tested a novel solution presented herein. This solution employs cationic and anionic exchange resins, which can exchange ions with the surrounding solution based on their concentration and affinity. However, since the i-ERs are not biocompatible with blood, we developed a specific hemodiafiltration circuit design. The regional anticoagulation was achieved by reducing the iCa concentration below 0.35 mmol/L, which represents the threshold associated with full anticoagulation in human blood [37]. First, we infused 4% sodium citrate (18 ml/min) at the blood inlet to achieve 5 mmol/L of citrate concentration which resulted in a significant reduction of iCa thus full anticoagulation of the blood. Subsequently, the blood passed through the hemofilters, before returning to the pig. The hemodiafiltrate effluent from the hemofilter, has a high flow, triple, compared to the blood flow so as to almost reach equilibrium. A small fraction of the flow (1170 mL/h) was discarded to achieve a neutral water balance.

Since we used an HDF flow three-times higher than the BF, the recirculating technique was mandatory. Otherwise, we would have needed 90 L/h of dialysis treatment. With the closed-loop circuit, we could reduce the WF to only 1.17 L/h (only 1.3% of the waste flow otherwise requested), making the technique more transferable to clinical practice in the coming years.

The first portion of treatment consists in citrate–calcium exchange with chloride and sodium, respectively, to maintain effective anticoagulation. Part of the hemodiafiltrate is then diverted to the rebalancing iERs capable of exchanging chloride and sodium ions with bicarbonate and hydrogen ions, respectively; these compounds led to carbonic acid formation, then split into water and carbon dioxide, the latter easily removable by membrane lung ventilation.

In addition to demonstrating the effectiveness of iER-based RCA in reducing iCa throughout the entire extracorporeal blood circuit, we also confirmed complete anticoagulation using KH–TEG analysis. No signs of clot formation were observed following citrate infusion and its subsequent removal (i.e., at points P1 and P9 in the circuit). Although the main determinant in citrate reduction is due to the net effect of the iER, the diffusion of citrate from the blood into the dialysate compartment may also have contributed in lowering the citrate systemic concentration. In contrast, KH–TEG analysis indicated the absence of systemic anticoagulation during the 2-h experiment (i.e., in the arterial blood sample). In addition to the effects due to the reduction of iCa concentrations in the circuit, we also observed a decrease in magnesium concentrations due to the non-selective action of iERs. Magnesium, like iCa, has been reported to contribute in activating several coagulating proteases [45].

Although full anticoagulation of the circuit, particularly at point P9 downstream of the hemodiafilter (after citrate removal), was maintained for 2 h, the efficiency of the iER cartridges used in the experiment declined over time. The cartridges achieved nearly 90% citrate removal within the first 15 min of treatment, approximately 70% at 60 min, and 50% at 120 min. Throughout the 2-h proof-of-concept experiment, no major complications occurred. During the first 60 min of iER-based RCA, no significant alterations in acid–base balance or electrolytes concentrations were recorded. However, in the second hour of treatment, direct and indirect signs of systemic citrate net overload were observed with non-compromised metabolism (e.g., arterial pH increased to 7.55, TCa accumulated to 15.5 mg/dL).

The pH increase during the second hour was mainly related to an unbalanced reduction in the efficiency of the different ion exchange resins. In particular, the first anionic resins reduced their function more significantly than the others, thus resulting in less citrate removal and chloride release, eventually leading to an increase in systemic citrate concentration and decrease in systemic chloride. These homeostasis alterations substantially differ from the citrate accumulation possibly occurring during CRRT undergoing RCA.

No major variations in blood gases, ventilatory parameters, hemodynamics, or other blood laboratory results were registered. Moreover, no signs of hemolysis were detected during the application of iER-based RCA technique.

Overall, our experiment suggests that the new custom-made iER circuit for high blood flow RCA effectively reduces iCa, providing complete regional anticoagulation without clinically relevant alterations in systemic parameters during the first hour of treatment. To ensure the safety and efficacy of longer-term treatments, it would be necessary to implement more capable cartridges, replace them more frequently, or reduce blood flow.

iER-based RCA offer several potential advantages: (1) it provides regional anticoagulation for high BF (up to 500 mL/min) without systemic anticoagulation, thereby avoiding severe or even fatal complications; (2) it overcomes the limited body citrate clearance, thus avoiding acid–base, electrolytes and metabolic disorders; (3) this technique could be used in low BF extracorporeal treatments for patients with liver failure, given that citrate metabolism primarily occurs in the liver; and (4) it may pave the way for new applications in other extracorporeal blood treatments, such as chronic dialysis, apheresis, and other organ support therapies, such as extracorporeal CO_2_ removal (ECCO_2_R) or liver substitution [46–48].

Notably, we envision the possibility of performing low-BF highly efficient ECCO_2_R (i.e., 300 mL/min of blood flow, with CO_2_ removal up to 100% of an adult production) without systemic anticoagulation by combining advanced CO_2_ removal techniques with iER-based RCA [29, 30]. This approach could expand the use of ECCO_2_R to a wide range of clinical scenarios, such as acute exacerbations of chronic obstructive pulmonary disease or as a bridge to lung transplant, without the currently high risk of bleeding, prohibitive for non-life-saving procedures.

However, several technical challenges and milestones must be addressed before clinical application can be considered. Currently, no ion-exchange resins have been approved for clinical use and, high-flow hemodiafilters suitable for integration with an ECCO_2_R circuit need to be developed. Moreover, the commercial scalability of such a system could be hindered by its intrinsic technological complexity. Despite these challenges, our experiment demonstrated the theoretical and short-term practical feasibility of this approach.

The study presents further limitations: (1) the swine model used may have negatively influenced the results, as pigs are known to be relatively hypercoagulable [49]. Also, only female swine have been enrolled in the study; (2) the experiment was limited to a 2-h period, so long-term efficacy and safety, both for the circuit (e.g., filter lifespan) and systemically (e.g., acid–base balance, electrolytes, metabolic, and bioumoral functions) were not assessed; (3) the i-ER-based RCA uses non-selective iERs. thus, even other electrolytes have been involved in the extracorporeal treatment; in particular the variation in magnesium and phosphate concentrations registered within 2 h experiment may be easily overcome by a custom-made titrated infusion; and (4) the study addressed the extracorporeal citrate clearance, but to assess the swine citrate clearance a control group without extracorporeal citrate removal would have required. Since the swine citrate clearance was not a primary aim of the study, following the 3Rs principles (Replacement, Reduction and Refinement), no control group was employed; furthermore, considering the global complexity of the experiment, we reduced the analyses to a minimum, looking only for citrate in the blood inside the extracorporeal circuit, before and after the dialysis treatment, and in the arterial blood, no dosage has been conducted inside the circuit, since we could estimate the citrate removal by measuring ions which were found to be simpler to dose by gas and electrolyte analysis (5) The impact on cytokine concentrations was not measured; thus no exploratory data are available on the inflammatory or anti-inflammatory effects of iER-based RCA; (6) The systemic use of heparin may have influenced the coagulation results. Although the use of KH–TEG helped mitigate this bias, other techniques like platelet function assays (e.g., aggregometry) were not evaluated; and (7) we only investigated the anticoagulation of the extracorporeal circuit without incorporating any extracorporeal organ support. The impact of the iER-based RCA on potential extracorporeal treatments will require further evaluation. (8) The study was designed with a fixed setup, assuming ideal conditions of exchange of the resins; thus, no resin has been changed, nor variation in flow. This proof of concept study allowed us to obtain as much data as possible on the efficiency of resins, finally providing fundamental information to design a long-term study.

## Conclusion

This study demonstrates that iER-based RCA is a feasible and effective technique for regional anticoagulation of extracorporeal blood flow up to 500 mL/min for 60 min without significant complications. However, further studies are needed to evaluate the long-term applications and effects on both the systemic system and the extracorporeal circuit.

## Supplementary Information

Additional file 1.

## Data Availability

Data were recorded on a local electronic worksheet. The data sets used and analyzed during the current study are available from the corresponding author upon reasonable request.

## Abbreviations

$$\alpha$$: $$\alpha$$ Angle
BF: Blood flow
ECCO_2_R: Extra corporeal CO_2_ removal
HDF: Hemodiafiltrate flow
iCa: Ionized calcium concentration
i-ER: Ion exchange resin
K: Kinetics time
KH-TEG: Heparinase Kaolin Thromboelastography
Ly30: Clot lysis 30 min after MA
MA: Maximum amplitude
ML: Membrane lung
R: Reaction time
RCA: Regional citrate anticoagulation
TCa: Total calcium concentration
UFH: UnFractionated heparin
WF: Waste flow
