# Supplementary material for: Citrate regional anticoagulation of 500 ml/min of extracorporeal blood flow: an experimental study
Source: Intensive Care Med Exp. 2025 Jun 24;13:64. doi: 10.1186/s40635-025-00771-7 (PMC12187626; doi:10.1186/s40635-025-00771-7)
Supplement: Supplementary file 1 — Additional file 1. [file 40635_2025_771_MOESM1_ESM.docx]

**Citrate regional anticoagulation of 500 ml/min of extracorporeal blood flow: an experimental study**

**Authors**

Sebastiano Maria Colombo^†1^, Luigi Vivona^†1,2^, Michele Battistin^3^, Vittorio Scaravilli^1,4^, Alessandro Galli^5^, Chiara Anzanello^6^, Elisa Cipriani^7^, Francesca Gori^1^, Serena Todaro^6^, Carlo Valsecchi^1^, Daniele Dondossola^8^, Anna Paola Marcello^9^, Andrea Carlin^6^, Antonio Pesenti^1,6^, Giacomo Grasselli^1,6^ and Alberto Zanella^1,6^

**Affiliations**

1. Fondazione IRCCS Ca' Granda Ospedale Maggiore Policlinico, Department of Anesthesia, Critical Care and Emergency. Milan, Italy
2. IRCCS MultiMedica San Giuseppe Hospital, Department of Anaesthesia and Intensive Care. Milan, Italy.
3. Fondazione IRCCS Ca' Granda Ospedale Maggiore Policlinico, Center for Preclinical Research. Milan, Italy
4. University of Milan, Department of Biomedical, Surgical and Dental Sciences. Milan, Italy
5. ASST Papa Giovanni XXIII, Dipartimento Emergenza, Urgenza e Area Critica. Bergamo, Italy
6. University of Milan, Department of Pathophysiology and Transplantation. Milan, Italy
7. Careggi University Hospital, Intensive Care Unit and Regional ECMO Referral Center, Florence, Italy
8. Fondazione IRCCS Ca' Granda Ospedale Maggiore Policlinico, Liver Transplant and General Surgery Unit. Milan, Italy
9. Fondazione IRCCS Ca' Granda Ospedale Maggiore Policlinico, Department of Medicine, Hematology - Fisiopatologia delle anemia. Milan, Italy
10. Fondazione IRCCS Ca' Granda Ospedale Maggiore Policlinico, Liver Transplant and General Surgery Unit. Milan, Italy

† These authors equally contributed and should be both considered first authors.

**SUPPLEMENTAL DIGITAL CONTENT**

- Additional methods
- Additional results

**ADDITIONAL METHODS**

**Animal preparation**

Six healthy domestic female pigs (41.00 ± 3.14 kg) underwent the study procedures. Twelve hours fasted swine were sedated (medetomidine 0.03 mg/Kg + Tiletamine/Zolazepam 5 mg/Kg through an intramuscular injection), intubated and mechanically ventilated. Afterward, total intravenous anesthesia was started (propofol 4-8 mg · kg^-1^ · h^-1^, medetomidine 3-12 mcg · kg^-1^ · h^-1^, pancuronium 0.2-0.6 mg · kg^-1^ · h^-1^). Analgesia was guaranteed through tramadol intravenous injection (2 mg/Kg).

Animals were then mechanically ventilated (Maquet Servo-I, Getinge, Rastatt, Germany) with a tidal volume of 10 mL/kg, PEEP of 5 cmH_2_O, FiO2 of 0.40, inspiratory to expiratory time ratio (I:E) of 1:2, and respiratory rate was set to maintain normocapnia (i.e., arterial partial pressure of carbon dioxide, PaCO2 = 35-45 mmHg). After administration of antibiotic prophylaxis with cefazolin (1 g, Teva Italia Srl, Milano, Italy), animals were instrumented with an arterial line in the right carotid (Seldicath 5 Fr, 8 cm, Prodimed, Le Plessis Bouchard, France), a central venous line (Triple Lumen, 7 Fr, 20 cm, Arrow, Reading, PA, US) and pulmonary artery catheters (132F5 Four Lumen, 5 Fr, 75 cm, Edwards Lifesciences, Irvine, CA, US) both in the left external jugular vein. Cystostomy was performed (Urinary catheter, straight, 2-way, 16 Fr, Coloplast SpA, Bologna, Italy). Following a parenteral injection of 150IU/kg of unfractionated heparin (UFH) (Epsoclar, Pfizer Srl, Latina, Italy), the right femoral vein and the right external jugular vein were cannulated (Single stage venous canula 18 Fr, 25 cm, Medtronic, Minneapolis, MN) and connected to a custom-made extracorporeal circuit optimized for i-ER-based RCA. A continuous infusion of UFH was provided targeting an activated clotting time > 300 seconds (GEM PCL Plus, Instrumentation Laboratory SpA Werfen, Milano, Italy).

**Animal monitoring and management**

During the entire experiment, the animals were monitored as follows:

- 4 leads electrocardiogram (ECG)
- Invasive Blood Pressure (IBP)
- Pulsatile peripheral oxygen saturation (SpO_2_) through the tail
- Diuresis
- Body core temperature (T)
- Ventilation parameters
- Activated Clotting Time (ACT)
- Arterial and mixed venous blood gas analysis

To ensure the correct working of the custom-made circuit, continuous pressure monitoring was provided along the different sections of the circuit (BP Amp and LabChart 8.0, AD Instruments, Dunedin, New Zealand).

During the entire experiment, a rehydrating solution was provided (Ringer Lactate, 3 mL · kg^-1^ · h^-1^, B. Braun, Melsungen, Germany). Euglycemia conditions were guaranteed, and antibiotics were repeated according to posology.

At the end of the experiment, animals were euthanized while still under deep sedation, with an injection of 40 mEq of potassium chloride.

**Ion exchange resins preparation**

The resins are high-capacity mixed bed ion exchange resins consisting of a mixture of a strong base anion resin and a strong acid cation resin (PMB 101-3 Pure Resin HSEDA Shangyu, Zhejiang, China). The treatment process started with the separation of the anionic component from the cationic one using a solution of NaCl 9%, which allows the separation of the two resins.

The anionic resins were charged with Cl^-^ or with HCO_3_^-^

- anionic resin with Cl^-^: 10 L of NaCl 9% solution, then rinsed with 10 L of distilled water and finally with 10 L of Bicarbonate Buffered Haemofiltration Solution 4 mmol/L potassium (MultiBic, Fresenius Medical Care, Bad Homburg, Germany).
- anionic resins with HCO_3_^-^: 20 L of NaHCO_3_ 1M solution, then rinsed with 10 L of distilled water and finally with 10 L of NaHCO_3_ 140 mM solution.

The cationic resins were charged with Na^+^/K^+^ or with H^+^.

- Cationic resins with Na^+^ and K^+^: 10 L of Na^+^ 1.4 M, K^+^ 0.04 M and Cl^-^ 1.44 M, then rinsed with 10 L of distilled water and finally with 5 L of MultiBic.
- Cationic resins with H^+^: 10 L of HCl 0.8M, then rinsed with 10 L of distilled water and finally with 5 L of 0.1 M HCl solution.

**Experimental design and measurements**

The sample size was based on our experience from previous experiments.

Upon completion of instrumentation, feasibility and safety of the i-ER-based RCA technique have been evaluated with a 2hrs proof of concept experiment. As previously described, flows across the circuit were set as follows:

- Blood flow (BF) 500 mL/min
- Hemodiafiltration flow (HDF) 1500 mL/min
- Waste flow (WF) 1170 mL/h, 19.5 mL/min
- Anionic resins with HCO_3_^-^ flow at 64 mL/min
- Cationic resins with H^+^ flow at 41 mL/min
- Calcium Chloride reinfusion according to Ca^++^
- Potassium Chloride reinfusion according to K^+^

These flow rates have been set according to previous elaborations through a mathematical model made in a calculation spreadsheet (Microsoft Excel, Office 365, Microsoft Corporation, Redmond, WA, US).

Nine different steps have been previously defined according to specific time points: Baseline (only blood flow, without active extracorporeal treatments), T2 (2 min), T8 (8 min), T15 (15 min), T30 (30 min), T45 (45-min), T60 (60-min), T90 (90-min), T120 (120 min).

Hemodynamics (i.e., heart rate, arterial pressures, core temperature, oxygen peripheral saturation), ventilatory parameters (i.e., minute ventilation, respiratory rate, tidal volume, peak pressure, plateau pressure, mean airway pressure), and circuit settings (i.e., flows, pressures) were recorded. In addition, drug, liquid infusions, electrolytes reinfusion, and glucose correction were also noted.

At each step, samples were withdrawn from arterial and circuit ports for gas analyses and ion concentrations (Ca^++^, Na^+^, Cl^-^) (ABL 800 gas analyzer; Radiometer, Copenhagen, Denmark). In addition, laboratory tests were also provided at specific time points to assess Magnesium and total Calcium concentrations (ADVIA Chemistry, Siemens Healthcare Diagnostics Inc, Tarrytown, NY), fibrinogen level according to Clauss method (ACL TOP 700 CTS, Instrumentation Laboratory, Bedford, MA), and complete blood count (XT-2000i, Sysmex, Kobe, Japan). Total proteins, hemolysis indicators like lactate dehydrogenase (ADVIA Chemistry, Siemens Healthcare Diagnostics Inc), and concentration of plasma-free hemoglobin were also estimated after instrumentation as well as at the end of the study.

**ADDITIONAL RESULTS**

**Regional Anticoagulation**

Complete results about calcium and citrate concentrations in the arterial blood (ART), blood inlet (P0), pre-filter blood (P1), and blood outlet (P9) are reported in Table S1.

**Electrolytes and Acid-Base balance**

Complete results about blood gas, electrolytes, and laboratory results in blood inlet (P0), pre-filter blood (P1), and blood outlet (P9) are reported in Table S2, S3, and S4, respectively.

**Systemic safety**

Complete results about hemodynamics and ventilatory settings are reported in Table S5.

**Table S1 – Calcium and citrate concentrations in arterial blood (ART), blood inlet (P0), pre-filter blood (P1), and blood outlet (P9)**

|  |  | **Baseline** | **T15** | | **T30** | | **T60** | | **T120** | **p - Value** | |
| --- | --- | --- | --- | --- | --- | --- | --- | --- | --- | --- | --- |
| **ART** | **iCa (mmol/L)** | 1.48 ± 0.04 | | 1.38 ± 0.10 | | 1.28 ± 0.09^&^ | | 1.16 ± 0.07^&$^ | 1.00 ± 0.17^&$#^ | | <.0001 |
|  | **TCa (mg/dL)** | 10.3 [10.1 - 10.8] | | 10.1 [9.9 - 10.8] | | 10.4 [10.1 - 10.6] | | 11.5 [11.0 - 12.5] | 15.5 [15.1 - 18.2] ^&$#@^ | | <.0001 |
|  | **TCa / iCa** | 1.76 [1.70 – 1.82] | | 1.89 [1.73 – 1.97] | | 2.04 [1.95 – 2.10] | | 2.48 [2.44 – 2.59] ^&$#^ | 4.00 [3.77– 4.73] ^&$#@^ | | <.0001 |
|  | **Citrate (mmol/L)** | 0.10 [0.06 – 0.11] | | 0.20 [0.17 – 0.32] | | 0.39 [0.33 – 0.53] | | 1.33 [1.17 – 1.39] ^&$#^ | 3.60 [3.11 – 3.94] ^&$#@^ | | <.0001 |
| **P0 *** | **iCa (mmol/L)** | 1.53 ± 0.11 | | 1.41 ± 0.10 | | 1.40 ± 0.10 | | 1.33 ± 0.05^&^ | 1.09 ± 0.14^&$#@^ | | <.0001 |
|  | **TCa (mg/dL)** | 10.3 [9.1 - 11.1] | | 10.3 [10.0 - 10.7] | | 10.6 [10.3 - 10.9] | | 11.7 [11.2 - 12.4] ^&^ | 15.1 [13.8 - 17.7] ^&$#@^ | | <.0001 |
|  | **Citrate (mmol/L)** | - | | - | | - | | - | - | | - |
| **P1^** | **iCa (mmol/L)** | 1.53 ± 0.11 | | 0.24 ± 0.02^&^ | | 0.25 ± 0.02^&^ | | 0.25 ± 0.02^&^ | 0.26 ± 0.04^&^ | | <.0001 |
|  | **TCa (mg/dL)** | 10.3 [9.1 - 11.1] | | - | | - | | - | - | | - |
|  | **Citrate (mmol/L)** | 0.10 [0.06 – 0.11] | | 7.22 [6.89 – 7.79] ^&^ | | 7.31 [7.24 – 7.81] ^&^ | | 7.85 [7.69 – 8.58] ^&^ | 11.38 [10.28 – 11.68] ^&$#@^ | | <.0001 |
| **P9** | **iCa (mmol/L)** | 1.53 ± 0.11 | | 0.19 ± 0.02^&^ | | 0.18 ± 0.03^&^ | | 0.19 ± 0.02^&^ | 0.23 ± 0.01^&$#@^ | | <.0001 |
|  | **TCa (mg/dL)** | 10.3 [9.1 - 11.1] | | 1.3 [1.2 - 1.7] ^&^ | | 1.4 [1.3 - 1.6] ^&^ | | 3.3 [3.2 – 3.4] ^&$#^ | 8.3 [7.9 – 8.9] ^&$#@^ | | <.0001 |
|  | **Citrate (mmol/L)** | 0.10 [0.06 – 0.11] | | 0.37 [0.30 – 0.56] ^&^ | | 0.89 [0.84 – 1.05] ^&$^ | | 2.38 [2.32 – 2.50] ^&$#^ | 5.75 [5.46 – 5.93] ^&$#@^ | | <.0001 |

Data are reported as median [IQR] or mean ± SD.

Samples were obtained from arterial blood (ART), blood inlet (P0), pre-filter blood (P1), and blood outlet (P9).

* Since P0 is before citrate infusion, citrate concentration in blood inlet (P0) = citrate concentration in arterial blood (ART).

^ Due to citrate infusion, TCa concentration was not obtained from P1.

Statistical analysis: ^&^ p < 0.05 Vs. Baseline, ^$^ p < 0.05 Vs. T15, ^#^ p < 0.05 Vs. T30, ^@^ p < 0.05 Vs. T60.

Abbreviations: pCO_2_, arterial carbon dioxide pressure; pO_2_, arterial oxygen pressure; Hb, hemoglobin; O_2_Hb, hemoglobin oxygen saturation; Hct, hematocrit; K^+^, potassium; Na^+^, sodium; Cl^-^, chloride; HCO3^-^, bicarbonates; Lac, lactate; Glu, glucose; BE, base excess; P, phosphorus; Mg, magnesium; iCa, ionized calcium (Ca^++^); TCa, total calcium.

**Table S2 - Blood gas, electrolytes, and laboratory results in blood inlet (P0)**

|  | **Baseline** | **T15** | | **T30** | | **T60** | | **T120** | **p - Value** | |
| --- | --- | --- | --- | --- | --- | --- | --- | --- | --- | --- |
| **pH** | 7.40 ± 0.00 | | 7.39 ± 0.08 | | 7.43 ± 0.06 | | 7.43 ± 0.05 | 7.46 ± 0.06^&$^ | | 0.0029 |
| **pCO_2_ (mmHg)** | 50.1 ± 0.1 | | 49.5 ± 13.1 | | 47.2 ± 10.5 | | 47.5 ± 9.7 | 45.4 ± 7.9 | | 0.1940 |
| **pO_2_ (mmHg)** | 41.6 ± 1.9 | | 39.8 ± 2.4 | | 37 ± 2.8^&^ | | 34.6 ± 4.2^&$^ | 32.5 ± 4.1^&$#^ | | <.0001 |
| **Hb (g/dL)** | 8.1 ± 0.7 | | 8.8 ± 1.1 | | 8.5 ± 2.5 | | 8.4 ± 1.6 | 8.9 ± 0.9 | | 0.9065 |
| **O_2_Hb (%)** | 47.9 ± 5.3 | | 55.7 ± 6.9 | | 51.3 ± 0.2 | | 49.7 ± 12 | 45.8 ± 10.3 | | 0.0592 |
| **Hct (%)** | 25.1 ± 2.1 | | 27.2 ± 3.2 | | 26.4 ± 7.7 | | 26.2 ± 4.8 | 27.5 ± 2.8 | | 0.8970 |
| **K^+^ (mmol/L)** | 4.0 ± 0.4 | | 3.9 ± 0.3 | | 4.0 ± 0.4 | | 4.0 ± 0.5 | 3.8 ± 0.5^&^ | | 0.0340 |
| **Na^+^ (mmol/L)** | 139.5 ± 4.9 | | 137.0 ± 1.4 | | 138.5 ± 4.0 | | 138.8 ± 4.7 | 134.3 ± 2.7^&#@^ | | 0.0088 |
| **Cl^-^ (mmol/L)** | 94.0 ± 5.7 | | 104.8 ± 4.8^&^ | | 103.7 ± 4.6^&^ | | 99.3 ± 5.0^$#^ | 93.5 ± 6.4^&$#@^ | | <.0001 |
| **HCO3^-^ (mmol/L)** | 30.5 ± 0.1 | | 29.3 ± 6.2^&^ | | 30.6 ± 6.5^&^ | | 31.1 ± 6.2^&$^ | 32.3 ± 7.4^&$#^ | | <.0001 |
| **Lac (mmol/L)** | 2.1 ± 0.1 | | 2.7 ± 0.5^&^ | | 2.4 ± 0.5 | | 2.7 ± 0.6^&^ | 2.9 ± 0.6^&#^ | | 0.0013 |
| **Glu (mg/dL)** | 137.5 ± 0.7 | | 124.2 ± 15.8 | | 127.7 ± 16.8 | | 122.3 ± 14.7 | 117.8 ± 16.6^#^ | | 0.0091 |
| **BE (mmol/L)** | 5.9 ± 2.5 | | 4.1 ± 6.8 | | 5.5 ± 7.0 | | 6.0 ± 6.6 | 7.5 ± 8.3^&$^ | | 0.0014 |
| **P (mg/dl)** | 7.53 ± 0.51 | | 6.30 ± 0.49^&^ | | 6.27 ± 0.54^&^ | | 6.25 ± 0.54^&^ | 6.82 ± 0.45^&$#@^ | | 0.0004 |
| **Mg (mg/dl)** | 1.62 [1.53 - 1.68] | | 1.32 [1.24 - 1.35] | | 1.23 [1.14 - 1.28] | | 1.23 [1.16 - 1.31] | 1.31 [1.26 - 1.41] | | 0.0576 |
| **iCa (mmol/L)** | 1.53 ± 0.11 | | 1.41 ± 0.10 | | 1.40 ± 0.10 | | 1.33 ± 0.05^&^ | 1.09 ± 0.14^&$#@^ | | <.0001 |
| **TCa (mg/dL)** | 10.3 [9.1 - 11.1] | | 10.3 [10.0 - 10.7] | | 10.6 [10.3 - 10.9] | | 11.7 [11.2 - 12.4] ^&^ | 15.1 [13.8 - 17.7] ^&$#@^ | | <.0001 |

Data are reported as median [IQR] or mean ± SD. Samples were obtained from the Blood inlet (P0).

Statistical analysis: ^&^ p < 0.05 Vs. Baseline, ^$^ p < 0.05 Vs. T15, ^#^ p < 0.05 Vs. T30, ^@^ p < 0.05 Vs. T60.

Abbreviations: pCO_2_, arterial carbon dioxide pressure; pO_2_, arterial oxygen pressure; Hb, hemoglobin; O_2_Hb, hemoglobin oxygen saturation; Hct, hematocrit; K^+^, potassium; Na^+^, sodium; Cl^-^, chloride; HCO3^-^, bicarbonates; Lac, lactate; Glu, glucose; BE, base excess; P, phosphorus; Mg, magnesium; iCa, ionized calcium (Ca^++^); TCa, total calcium.

**Table S3 - Blood gas, electrolytes, and laboratory results in Pre-filter blood (P1)**

|  | **Baseline *** | **T15** | **T30** | **T60** | **T120** | **p - Value** |
| --- | --- | --- | --- | --- | --- | --- |
| **pH** | 7.40 ± 0.00 | 7.37 ± 0.07^&^ | 7.40 ± 0.05 | 7.41 ± 0.06^$^ | 7.43 ± 0.06^$^ | 0.0012 |
| **pCO_2_ (mmHg)** | 50.1 ± 0.1 | 49.5 ± 12.2 | 48.1 ± 10.3 | 47.4 ± 9.9 | 45.9 ± 9.2 | 0.0871 |
| **pO_2_ (mmHg)** | 41.6 ± 1.9 | 41.2 ± 3.6 | 37.4 ± 2.9^&$^ | 35.9 ± 4.2^&$^ | 32.5 ± 3.3^&$#@^ | <.0001 |
| **Hb (g/dL)** | 8.1 ± 0.7 | 8.1 ± 1.1 | 8.7 ± 0.8 | 8.9 ± 1.1 | 8.8 ± 0.8 | 0.0739 |
| **O_2_Hb (%)** | 47.9 ± 5.3 | 61.6 ± 11.1^&^ | 54.1 ± 7.4^&^ | 49.9 ± 12.8^$^ | 46.4 ± 8.3^$^ | 0.0084 |
| **Hct (%)** | 25.1 ± 2.1 | 25.0 ± 3.4 | 27.0 ± 2.4 | 27.7 ± 3.5 | 27.4 ± 2.4 | 0.0649 |
| **K^+^ (mmol/L)** | 4.0 ± 0.4 | 3.7 ± 0.3^&^ | 3.7 ± 0.3^&^ | 3.7 ± 0.4^&^ | 3.4 ± 0.4^&$#@^ | 0.0106 |
| **Na^+^ (mmol/L)** | 139.5 ± 4.9 | 142.7 ± 2.1 | 145.0 ± 4.1 | 143.7 ± 3.9 | 140.5 ± 1.2 | 0.0570 |
| **Cl^-^ (mmol/L)** | 94.0 ± 5.7 | 97.8 ± 2.9 | 99.2 ± 6.4 | 96.2 ± 5.5 | 89.0 ± 7.7^&$#@^ | <.0001 |
| **HCO3^-^ (mmol/L)** | 30.5 ± 0.1 | 28.0 ± 5.5 | 29.3 ± 6.1 | 29.9 ± 6.0^$^ | 30.6 ± 7.1^$^ | 0.0005 |
| **Lac (mmol/L)** | 2.1 ± 0.1 | 2.5 ± 0.4^&^ | 2.2 ± 0.4 | 2.5 ± 0.5^&^ | 2.7 ± 0.6^&#^ | 0.0258 |
| **Glu (mg/dL)** | 137.5 ± 0.7 | 116.5 ± 14.0^&^ | 115.5 ± 15.1^&^ | 121.0 ± 16.4^&^ | 111.5 ± 14.3^&@^ | 0.0444 |
| **BE (mmol/L)** | 5.9 ± 2.5 | 2.5 ± 5.8^&^ | 4.0 ± 6.4 | 4.7 ± 6.4^$^ | 5.7 ± 7.9^$^ | 0.0030 |
| **P (mg/dl)** | 7.53 ± 0.51 | - | - | - | - | - |
| **Mg (mg/dl)** | 1.62 [1.53 - 1.68] | - | - | - | - | - |
| **iCa (mmol/L)** | 1.53 ± 0.11 | 0.24 ± 0.02^&^ | 0.25 ± 0.02^&^ | 0.25 ± 0.02^&^ | 0.26 ± 0.04^&^ | <.0001 |
| **TCa (mg/dL)** | 10.3 [9.1 - 11.1] | - | - | - | - | - |

Data are reported as median [IQR] or mean ± SD. Samples were obtained from the Pre-filter blood (P1).

* At Baseline blood inlet (P0) = Pre-filter blood (P1) since no extracorporeal treatment was running.

Statistical analysis: ^&^ p < 0.05 Vs. Baseline, ^$^ p < 0.05 Vs. T15, ^#^ p < 0.05 Vs. T30, ^@^ p < 0.05 Vs. T60.

Abbreviations: pCO_2_, arterial carbon dioxide pressure; pO_2_, arterial oxygen pressure; Hb, hemoglobin; O_2_Hb, hemoglobin oxygen saturation; Hct, hematocrit; K^+^, potassium; Na^+^, sodium; Cl^-^, chloride; HCO3^-^, bicarbonates; Lac, lactate; Glu, glucose; BE, base excess; P, phosphorus; Mg, magnesium; iCa, ionized calcium (Ca^++^); TCa, total calcium.

**Table S4 - Blood gas, electrolytes, and laboratory results in Blood outlet (P9)**

|  | **Baseline *** | **T15** | | **T30** | | **T60** | | **T120** | | **p - Value** | | |
| --- | --- | --- | --- | --- | --- | --- | --- | --- | --- | --- | --- | --- |
| **pH** | 7.40 ± 0.00 | | 7.57 ± 0.04^&^ | | 7.57 ± 0.04^&^ | | 7.58 ± 0.03^&^ | | 7.58 ± 0.06^&^ | | 0.0024 |  |
| **pCO_2_ (mmHg)** | 50.1 ± 0.1 | | 34.2 ± 2.7^&^ | | 34.8 ± 2.4^&^ | | 33.5 ± 2.2^&^ | | 33.4 ± 3.7^&^ | | <.0001 |  |
| **pO_2_ (mmHg)** | 41.6 ± 1.9 | | 147.1 ± 68.1^&^ | | 108.9 ± 47.4^&^ | | 109.6 ± 64.9^&^ | | 76.3 ± 29.8^&^ | | <.0001 |  |
| **Hb (g/dL)** | 8.1 ± 0.7 | | 8.8 ± 1.6 | | 9.3 ± 1.3 | | 10.0 ± 1.9 | | 8.9 ± 1.1 | | 0.1812 |  |
| **O_2_Hb (%)** | 47.9 ± 5.3 | | 97.5 ± 0.4^&^ | | 97.6 ± 2.3^&^ | | 96.3 ± 3.2^&^ | | 94.4 ± 2.5^&^ | | <.0001 |  |
| **Hct (%)** | 25.1 ± 2.1 | | 27.3 ± 4.8 | | 28.7 ± 3.9 | | 30.9 ± 5.7 | | 27.6 ± 3.2 | | 0.1889 |  |
| **K^+^ (mmol/L)** | 4.0 ± 0.4 | | 3.5 ± 0.1^&^ | | 3.4 ± 0.2^&^ | | 3.4 ± 0.3^&^ | | 3.3 ± 0.3^&$^ | | 0.0125 |  |
| **Na^+^ (mmol/L)** | 139.5 ± 4.9 | | 140.4 ± 4.4 | | 136.2 ± 2.0 | | 135.2 ± 4.8^$^ | | 129.7 ± 2^&$#@^ | | 0.0002 |  |
| **Cl^-^ (mmol/L)** | 94.0 ± 5.7 | | 103.8 ± 5.4^&^ | | 99.2 ± 4.3^&$^ | | 94.2 ± 5.4^&$#^ | | 85.8 ± 8.7^&$#@^ | | <.0001 |  |
| **HCO3^-^ (mmol/L)** | 30.5 ± 0.1 | | 31.3 ± 4.2 | | 32.1 ± 3.2 | | 31.8 ± 2.7 | | 31.9 ± 4.1 | | 0.6165 |  |
| **Lac (mmol/L)** | 2.1 ± 0.1 | | 1.9 ± 0.3 | | 2.0 ± 0.3 | | 2.2 ± 0.4^$^ | | 2.6 ± 0.6^&$#@^ | | <.0001 |  |
| **Glu (mg/dL)** | 137.5 ± 0.7 | | 91.4 ± 9.5^&^ | | 102.2 ± 12.6^&$^ | | 113.5 ± 10.1^&$#^ | | 111.2 ± 13.5^&$#^ | | <.0001 |  |
| **BE (mmol/L)** | 5.9 ± 2.5 | | 7.5 ± 4.5^&^ | | 8.7 ± 3.4^&^ | | 8.6 ± 2.7^&^ | | 8.6 ± 4.4^&^ | | 0.0436 |  |
| **P (mg/dl)** | 7.53 ± 0.51 | | 3.10 ± 0.29^&^ | | 3.98 ± 0.46^&$^ | | 4.75 ± 0.42^&$#^ | | 5.33 ± 0.29^&$#@^ | | <.0001 |  |
| **Mg (mg/dl)** | 1.62 [1.53 - 1.68] | | 0.45 [0.40 – 0.47] ^&^ | | 0.56 [0.49 – 0.65] ^&$^ | | 0.88 [0.79 – 0.97] ^&$#^ | | 0.96 [0.92 - 1.00] ^&$#@^ | | <.0001 |  |
| **iCa (mmol/L)** | 1.53 ± 0.11 | | 0.19 ± 0.02^&^ | | 0.18 ± 0.03^&^ | | 0.19 ± 0.02^&^ | | 0.23 ± 0.01^&$#@^ | | <.0001 |  |
| **TCa (mg/dL)** | 10.3 [9.1 - 11.1] | | 1.3 [1.2 - 1.7] ^&^ | | 1.4 [1.3 - 1.6] ^&^ | | 3.3 [3.2 – 3.4] ^&$#^ | | 8.3 [7.9 – 8.9] ^&$#@^ | | <.0001 |  |

Data are reported as median [IQR] or mean ± SD. Samples were obtained from the Blood outlet (P9).

* At Baseline blood inlet (P0) = Pre-filter blood (P9) since no extracorporeal treatment was running.

Statistical analysis: ^&^ p < 0.05 Vs. Baseline, ^$^ p < 0.05 Vs. T15, ^#^ p < 0.05 Vs. T30, ^@^ p < 0.05 Vs. T60.

Abbreviations: pCO_2_, arterial carbon dioxide pressure; pO_2_, arterial oxygen pressure; Hb, hemoglobin; O_2_Hb, hemoglobin oxygen saturation; Hct, hematocrit; K^+^, potassium; Na^+^, sodium; Cl^-^, chloride; HCO3^-^, bicarbonates; Lac, lactate; Glu, glucose; BE, base excess; P, phosphorus; Mg, magnesium; iCa, ionized calcium (Ca^++^); TCa, total calcium.

**Table S5 - Hemodynamics and ventilatory settings**

|  | **Baseline** | **T15** | **T30** | **T60** | **T120** | **p-Value** |
| --- | --- | --- | --- | --- | --- | --- |
| **HR (bpm)** | 106 [95.5 - 123] | 114.5 [82 - 125.25] | 115 [80.5 - 128] | 118.5 [90.25 - 127.5] | 118.5 [103 - 124.5] | 0.3187 |
| **SBP (mmHg)** | 108.5 [99.75 - 125] | 100 [94 - 111.25] | 91 [88.75 - 102.25]^&^ | 93.5 [90.75 - 103] | 92 [87 - 97.5]^&^ | 0.0127 |
| **DBP (mmHg)** | 70 [67.75 - 97.5] | 72.5 [57.25 - 80] | 59.5 [55.25 - 76] | 63 [58 - 71.75] | 60 [56.5 - 74.5] | 0.0975 |
| **MAP (mmHg)** | 84 [83 - 99] | 83 [67.5 - 90.85] | 71 [66 -82] | 75 [69 - 78] | 71 [66 - 79.5] | 0.1046 |
| **SpO_2_ (%)** | 100 [100 - 100] | 100 [100 - 100] | 100 [99 - 100] | 100 [99 - 100] | 100 [99 - 100] | 0.4307 |
| **Temp (°C)** | 36.7 [36.4 - 37.3] | 37.5 [36.7 - 37.95]^&^ | 37.4 [36.9 - 37.8]^&^ | 37.6 [37.3 - 38]^&^ | 37.8 [37.7 - 38.1]^&$#^ | < 0.001 |
| **MV (L/min)** | 7.9 [7.1 - 8.1] | 7.8 [7.4 - 8.3] | 7.8 [7.4 - 8.3] | 7.8 [7.4 - 8.3] | 7.8 [6.6 - 8.3] | 0.6842 |
| **FiO_2_ (%)** | 0.40 [0.40 - 0.40] | 0.40 [0.40 - 0.40] | 0.40 [0.40 - 0.40] | 0.40 [0.40 - 0.40] | 0.40 [0.40 - 0.40] | 1.000 |
| **RR (bpm)** | 19 [15 - 22] | 18 [16 - 22] | 18 [16 - 22] | 18 [16 - 22] | 16 [15 - 21] | 0.3359 |
| **TV (mL)** | 415 [371 - 498] | 413 [371 - 498] | 413 [371 - 498] | 413 [371 - 498] | 413 [373 - 498] | 0.6864 |
| **PEEP (cmH_2_O)** | 5 [5 - 5] | 5 [5 - 5] | 5 [5 - 5] | 5 [5 - 5] | 5 [5 - 5] | 1.000 |
| **PPk (cmH_2_O)** | 28 [26 - 30] | 28 [25 - 29] | 27 [25 - 29] | 28 [25 - 29] | 28 [25 - 29] | 0.7123 |
| **PPl (cmH_2_O)** | 20 [18 - 21] | 19 [17 - 21] | 20 [17 - 21] | 21 [17 - 21] | 19 [17 - 21] | 0.6297 |
| **mPaw (cmH_2_O)** | 10 [10 - 10] | 10 [10 - 10] | 10 [10 - 10] | 10 [10 - 10] | 10 [10 - 10] | 1.000 |

Data are reported as median [IQR] or mean ± SD.

Statistical analysis: ^&^ p < 0.05 Vs. Baseline, ^$^ p < 0.05 Vs. T15, ^#^ p < 0.05 Vs. T30, ^@^ p < 0.05 Vs. T60.

Abbreviations: HR, heart rate; SBP, systolic blood pressure; DBP, diastolic blood pressure; MAP, mean arterial pressure; SpO_2_, Pulsatile peripheral oxygen saturation; Temp, core body temperature; MV, minute ventilation; FiO_2_, fraction of inspired oxygen; RR, respiratory rate; TV, tidal volume; PEEP, positive end-expiratory pressure; PPk, Peak of inspiratory pressure; PPl, Plateau pressure; mPaw, mean airway pressure.

**Table S6 - Blood gas and electrolytes in hemodiafilter circuit (P2)**

|  | **Baseline** | **T15** | **T30** | | **T60** | | **T120** | | **p - Value** | | |
| --- | --- | --- | --- | --- | --- | --- | --- | --- | --- | --- | --- |
| **pH** | - | 7.58 ± 0.12 | | 7.66 ± 0.08 | | 7.63 ± 0.04 | | 7.65 ± 0.02 | | 0.2166 | |
| **pCO_2_ (mmHg)** | - | 43.9 ± 13.8 | | 36.7 ± 4.8 | | 39.3 ± 2.9 | | 38.3 ± 2.6 | | 0.3319 | |
| **pO_2_ (mmHg)** | - | 161.7 ± 11.8 | | 160.2 ± 13.5 | | 155.6 ± 6.8 | | 140.2 ± 35.6 | | 0.2906 | |
| **K^+^ (mmol/L)** | - | 3.5 ± 0.2 | | 3.5 ± 0.2 | | 3.4 ± 0.4 | | 3.3 ± 0.4^$#^ | | 0.0061 | |
| **Na^+^ (mmol/L)** | - | 138.5 ± 2.1 | | 137.7 ± 1.2 | | 134.0 ± 3.0^$#^ | | 129.3 ± 2.4^$#@^ | | < .0001 | |
| **Cl^-^ (mmol/L)** | - | 101.8 ± 1.0 | | 98.7 ± 1.4 | | 92.2 ± 2.2 | | 90.3 ± 16.6 | | | 0.1315 |
| **HCO3^-^ (mmol/L)** | - | 40.1 ± 2.2 | | 42.0 ± 3.9 | | 41.7 ± 1.4 | | 42.9 ± 1.3 | | 0.3103 | |
| **Lac (mmol/L)** | - | 1.8 ± 0.4 | | 1.9 ± 0.3 | | 2.1 ± 0.5 | | 2.6 ± 0.6^$#@^ | | < .0001 | |
| **Glu (mg/dL)** | - | 110.7 ± 13.8 | | 116.2 ± 13.2 | | 129.6 ± 14.7^$^ | | 126.0 ± 16.7^$#^ | | 0.0004 | |
| **BE (mmol/L)** | - | 15.8 ± 3.2 | | 18.2 ± 4.0 | | 17.7 ± 1.6 | | 19.6 ± 0.7 | | 0.2513 | |
| **P (mg/dl)** | - | - | | - | | - | | - | | |  |
| **Mg (mg/dl)** | - | - | | - | | - | | - | | |  |
| **iCa (mmol/L)** | - | 0.21 ± 0.02 | | 0.19 ± 0.01 | | 0.19 ± 0.01 | | 0.22 ± 0.02^#@^ | | 0.0114 | |
| **TCa (mg/dL)** | - | - | | - | | - | | - | | |  |

Data are reported as median [IQR] or mean ± SD. Samples were obtained from the circuit (P2).

Statistical analysis: ^&^ p < 0.05 Vs. Baseline, ^$^ p < 0.05 Vs. T15, ^#^ p < 0.05 Vs. T30, ^@^ p < 0.05 Vs. T60.

Abbreviations: pCO_2_, arterial carbon dioxide pressure; pO_2_, arterial oxygen pressure; K^+^, potassium; Na^+^, sodium; Cl^-^, chloride; HCO3^-^, bicarbonates; Lac, lactate; Glu, glucose; BE, base excess; P, phosphorus; Mg, magnesium; iCa, ionized calcium (Ca^++^); TCa, total calcium.

**Table S7 - Blood gas and electrolytes in hemodiafilter circuit (P3)**

|  | **Baseline** | **T15** | **T30** | | **T60** | | **T120** | | **p - Value** | |
| --- | --- | --- | --- | --- | --- | --- | --- | --- | --- | --- |
| **pH** | - | 7.54 ± 0.12 | | 7.64 ± 0.05^$^ | | 7.64 ± 0.03 | | 7.64 ± 0.02^$^ | | 0.0212 |
| **pCO_2_ (mmHg)** | - | 45.7 ± 10.9 | | 38.3 ± 4.3 | | 38.1± 2.9 | | 38.5 ± 1.8 | | 0.0680 |
| **pO_2_ (mmHg)** | - | 155.8 ± 8.6 | | 158.0 ± 10.4 | | 159.0 ± 10.5 | | 153.3 ± 15.2 | | 0.3117 |
| **K^+^ (mmol/L)** | - | 3.8 ± 0.1 | | 3.6 ± 0.1 | | 3.5 ± 0.3 | | 3.3 ± 0.3 | | 0.0002 |
| **Na^+^ (mmol/L)** | - | 141.5 ± 1.4 | | 140.2 ± 1.9^$^ | | 136.2 ± 1.0^$#^ | | 132.5 ± 1.6^$#@^ | | < .0001 |
| **Cl^-^ (mmol/L)** | - | 105.8 ± 2.1 | | 102.2 ± 1.6^$^ | | 96.3 ± 1.9^$#^ | | 85.5 ± 1.9^$#@^ | | < .0001 |
| **HCO3^-^ (mmol/L)** | - | 38.7 ± 3.5 | | 41.6 ± 1.2^$^ | | 41.2 ± 2.2^$^ | | 42.5 ± 1.9^$^ | | 0.0020 |
| **Lac (mmol/L)** | - | 1.7 ± 0.4 | | 1.9 ± 0.4 | | 2.2 ± 0.4^$^ | | 2.7 ± 0.6^$#@^ | | < .0001 |
| **Glu (mg/dL)** | - | 114.3 ± 15.4 | | 114.8 ± 10.1 | | 127.0 ± 14.9^$#^ | | 123.3 ± 14.8 | | 0.0030 |
| **BE (mmol/L)** | - | 14.1 ± 4.4 | | 18.0 ± 1.3^$^ | | 17.6 ± 2.2^$^ | | 18.7 ± 1.9^$^ | | 0.0060 |
| **P (mg/dl)** | - | 3.38 ± 0.61 | | 4.25 ± 0.56^$^ | | 5.00 ± 0.49^$#^ | | 5.45 ± 0.67^$#^ | | < .0001 |
| **Mg (mg/dl)** | - | 0.18 [0.15 – 0.21] | | 0.36 [0.31 – 0.44]^$^ | | 0.63 [0.58 – 0.77]^$#^ | | 0.70 [0.65 – 0.78]^$#^ | | < .0001 |
| **iCa (mmol/L)** | - | 0.17 ± 0.03 | | 0.14 ± 0.03 | | 0.17 ± 0.02 | | 0.22 ± 0.01^$#@^ | | < .0001 |
| **TCa (mg/dL)** | - | 0.10 [0.10 – 0.14] | | 0.28 [0.18 – 0.39] | | 2.27 [2.05 – 2.63]^$#^ | | 8.10 [7.36 – 8.43]^$#@^ | | < .0001 |

Data are reported as median [IQR] or mean ± SD. Samples were obtained from the circuit (P3).

Statistical analysis: ^&^ p < 0.05 Vs. Baseline, ^$^ p < 0.05 Vs. T15, ^#^ p < 0.05 Vs. T30, ^@^ p < 0.05 Vs. T60.

Abbreviations: pCO_2_, arterial carbon dioxide pressure; pO_2_, arterial oxygen pressure; K^+^, potassium; Na^+^, sodium; Cl^-^, chloride; HCO3^-^, bicarbonates; Lac, lactate; Glu, glucose; BE, base excess; P, phosphorus; Mg, magnesium; iCa, ionized calcium (Ca^++^); TCa, total calcium.

**Table S8 - Blood gas and electrolytes in hemodiafilter circuit (P6)**

|  | **Baseline** | **T15** | **T30** | | **T60** | | **T120** | | **p - Value** | | |
| --- | --- | --- | --- | --- | --- | --- | --- | --- | --- | --- | --- |
| **pH** | - | 6.51 ± 0.12 | | 6.39 ± 0.03 | | 6.40 ± 0.10 | | 6.52 ± 0.24 | | | 0.4176 |
| **pCO_2_ (mmHg)** | - | 583.3 ± 112.8 | | 636.0 ± 25.2 | | 532.7 ± 240.8 | | 515.3 ± 219.5 | | | 0.7283 |
| **pO_2_ (mmHg)** | - | 94.5 ± 16.7 | | 82.7 ± 12.4 | | 102.3 ± 32.9 | | 92.1 ± 30.1 | | | 0.6775 |
| **K^+^ (mmol/L)** | - | 0.1 ± 0.1 | | 1.7 ± 0.5^$^ | | 2.1 ± 0.1^$^ | | 2.1 ± 0.2^$^ | | | < .0001 |
| **Na^+^ (mmol/L)** | - | 94.2 ± 2.3 | | 91.0 ± 2.2^$^ | | 84.5 ± 7.0 | | 88.0 ± 4.0 | | | 0.0069 |
| **Cl^-^ (mmol/L)** | - | 39.0 ± 2.3 | | 41.8 ± 1.0 | | 43.5 ± 3.3 | | 35.0 ± 2.4^#@^ | | | 0.0026 |
| **HCO3^-^ (mmol/L)** | - | 45.2 ± 5.8 | | 37.3 ± 2.7^$^ | | 30.1 ± 9.7 | | 40.5 ± 6.8 | | | 0.0383 |
| **Lac (mmol/L)** | - | 0.1 ± 0.1 | | 0.2 ± 0.1 | | 1.1 ± 0.3^$#^ | | 2.5 ± 0.5^$#@^ | | | < .0001 |
| **Glu (mg/dL)** | - | 14.5 ± 8.0 | | 82.3 ± 18.0^$^ | | 124.0 ± 9.4^$#^ | | 145.3 ± 3.3^$#^ | | | < .0001 |
| **BE (mmol/L)** | - | 3.0 ± 10.4 | | -8.5 ± 3.8 | | -11.7 ± 8.5 | | 2.2 ± 10.3 | | 0.1814 | |
| **P (mg/dl)** | - | - | | - | | - | | - | | |  |
| **Mg (mg/dl)** | - | - | | - | | - | | - | | |  |
| **iCa (mmol/L)** | - | 0.09 ± 0.01 | | 0.11 ± 0.02 | | 0.09 ± 0.01 | | 0.14 ± 0.02^$@^ | | 0.0031 | |
| **TCa (mg/dL)** | - | - | | - | | - | | - | | |  |

Data are reported as median [IQR] or mean ± SD. Samples were obtained from the circuit (P6).

Statistical analysis: ^&^ p < 0.05 Vs. Baseline, ^$^ p < 0.05 Vs. T15, ^#^ p < 0.05 Vs. T30, ^@^ p < 0.05 Vs. T60.

Abbreviations: pCO_2_, arterial carbon dioxide pressure; pO_2_, arterial oxygen pressure; K^+^, potassium; Na^+^, sodium; Cl^-^, chloride; HCO3^-^, bicarbonates; Lac, lactate; Glu, glucose; BE, base excess; P, phosphorus; Mg, magnesium; iCa, ionized calcium (Ca^++^); TCa, total calcium.

**Table S9 - Blood gas and electrolytes in hemodiafilter circuit (P7)**

|  | **Baseline** | **T15** | **T30** | | **T60** | | **T120** | | **p - Value** | | |
| --- | --- | --- | --- | --- | --- | --- | --- | --- | --- | --- | --- |
| **pH** | - | 7.26 ± 0.11 | | 7.23 ± 0.03 | | 7.18 ± 0.09 | | 7.16 ± 0.06 | | 0.0510 | |
| **pCO_2_ (mmHg)** | - | 79.3 ± 18.9 | | 82.2 ± 7.2 | | 90.5 ± 16.0 | | 96.3 ± 11.9 | | 0.0817 | |
| **pO_2_ (mmHg)** | - | 160.2 ± 8.0 | | 154.8 ± 10.7 | | 153.2 ± 10.8 | | 146.8 ± 8.9^$^ | | 0.0364 | |
| **K^+^ (mmol/L)** | - | 3.5 ± 0.1 | | 3.4 ± 0.2 | | 3.4 ± 0.2 | | 3.3 ± 0.3^$^ | | 0.0082 | |
| **Na^+^ (mmol/L)** | - | 138.0 ± 3.0 | | 136.0 ± 2.0 | | 132.5 ± 2.2^$^ | | 128.7 ± 1.9^$#@^ | | < .0001 | |
| **Cl^-^ (mmol/L)** | - | 103.0 ± 2.4 | | 98.3 ± 2.0^$^ | | 91.3 ± 2.8^$#^ | | 81.3 ± 2.7^$#@^ | | < .0001 | |
| **HCO3^-^ (mmol/L)** | - | 33.8 ± 1.8 | | 32.8 ± 0.8 | | 32.0 ± 1.9 | | 32.7 ± 1.9 | | 0.1360 | |
| **Lac (mmol/L)** | - | 1.6 ± 0.3 | | 1.8 ± 0.4 | | 2.0 ± 0.4^$^ | | 2.7 ± 0.6^$#@^ | | < .0001 | |
| **Glu (mg/dL)** | - | 109.0 ± 13.6 | | 113.8 ± 11.7 | | 124.5 ± 15.2^$#^ | | 125.5 ± 14.7^$#^ | | 0.0002 | |
| **BE (mmol/L)** | - | 6.6 ± 3.1 | | 5.6 ± 0.9 | | 4.2 ± 3.3 | | 4.9 ± 2.5 | | 0.2472 | |
| **P (mg/dl)** | - | - | | - | | - | | - | | |  |
| **Mg (mg/dl)** | - | - | | - | | - | | - | | |  |
| **iCa (mmol/L)** | - | 0.15 ± 0.01 | | 0.14 ± 0.01^$^ | | 0.15 ± 0.01 | | 0.22 ± 0.02^$#@^ | | < .0001 | |
| **TCa (mg/dL)** | - | - | | - | | - | | - | | |  |

Data are reported as median [IQR] or mean ± SD. Samples were obtained from the circuit (P7).

Statistical analysis: ^&^ p < 0.05 Vs. Baseline, ^$^ p < 0.05 Vs. T15, ^#^ p < 0.05 Vs. T30, ^@^ p < 0.05 Vs. T60.

Abbreviations: pCO_2_, arterial carbon dioxide pressure; pO_2_, arterial oxygen pressure; K^+^, potassium; Na^+^, sodium; Cl^-^, chloride; HCO3^-^, bicarbonates; Lac, lactate; Glu, glucose; BE, base excess; P, phosphorus; Mg, magnesium; iCa, ionized calcium (Ca^++^); TCa, total calcium.

**Table S10 - Blood gas and electrolytes in hemodiafilter circuit (P8)**

|  | **Baseline** | **T15** | **T30** | | **T60** | | **T120** | | **p - Value** | | |
| --- | --- | --- | --- | --- | --- | --- | --- | --- | --- | --- | --- |
| **pH** | - | 7.80 ± 0.34 | | 7.94 ± 0.03 | | 7.93 ± 0.03 | | 7.91 ± 0.03 | | 0.5510 | |
| **pCO_2_ (mmHg)** | - | 39.1 ± 34.9 | | 25.2 ± 2.5 | | 24.5 ± 2.0 | | 25.2 ± 1.4 | | 0.4462 | |
| **pO_2_ (mmHg)** | - | 294.3 ± 59.7 | | 322.3 ± 31.8 | | 317.8 ± 48.7 | | 351.2 ± 43.3 | | 0.1173 | |
| **K^+^ (mmol/L)** | - | 3.5 ± 0.1 | | 3.4 ± 0.2 | | 3.4 ± 0.2 | | 3.3 ± 0.3^$^ | | 0.0302 | |
| **Na^+^ (mmol/L)** | - | 138.0 ± 1.8 | | 136.0 ± 2.2 | | 132.5 ± 1.9^$#^ | | 129.0 ± 1.8^$#@^ | | < .0001 | |
| **Cl^-^ (mmol/L)** | - | 102.2 ± 1.3 | | 98.3 ± 1.4^$^ | | 92.7 ± 1.4^$#^ | | 82.2 ± 1.3^$#@^ | | < .0001 | |
| **HCO3^-^ (mmol/L)** | - | 54.3 ± 10.9 | | 58.3 ± 3.4 | | 55.3 ± 2.7 | | 55.2 ± 3.1 | | 0.6194 | |
| **Lac (mmol/L)** | - | 1.6 ± 0.3 | | 1.9 ± 0.4 | | 2.1 ± 0.4^$^ | | 2.7 ± 0.7^$#@^ | | < .0001 | |
| **Glu (mg/dL)** | - | 105.5 ± 13.9 | | 115.8 ± 13.3^$^ | | 125.8 ± 14.8^$#^ | | 127.5 ± 14.8^$#^ | | < .0001 | |
| **BE (mmol/L)** | - | 25.4 ± 12.2 | | 30.7 ± 2.0 | | 28.9 ± 1.8 | | 29.1 ± 2.5 | | 0.6008 | |
| **P (mg/dl)** | - | - | | - | | - | | - | | |  |
| **Mg (mg/dl)** | - | - | | - | | - | | - | | |  |
| **iCa (mmol/L)** | - | 0.14 ± 0.02 | | 0.12 ± 0.01 | | 0.14 ± 0.01^#^ | | 0.21 ± 0.01^$#@^ | | < .0001 | |
| **TCa (mg/dL)** | - | - | | - | | - | | - | | |  |

Data are reported as median [IQR] or mean ± SD. Samples were obtained from the circuit (P8).

Statistical analysis: ^&^ p < 0.05 Vs. Baseline, ^$^ p < 0.05 Vs. T15, ^#^ p < 0.05 Vs. T30, ^@^ p < 0.05 Vs. T60.

Abbreviations: pCO_2_, arterial carbon dioxide pressure; pO_2_, arterial oxygen pressure; K^+^, potassium; Na^+^, sodium; Cl^-^, chloride; HCO3^-^, bicarbonates; Lac, lactate; Glu, glucose; BE, base excess; P, phosphorus; Mg, magnesium; iCa, ionized calcium (Ca^++^); TCa, total calcium.
